# Conditioned media from adipose stromal cells limit lipopolysaccharide-induced lung injury, endothelial hyperpermeability and apoptosis

**DOI:** 10.1186/s12967-015-0422-3

**Published:** 2015-02-21

**Authors:** Hongyan Lu, Christophe Poirier, Todd Cook, Dmitry O Traktuev, Stephanie Merfeld-Clauss, Benjamin Lease, Irina Petrache, Keith L March, Natalia V Bogatcheva

**Affiliations:** Division of Cardiology, Indiana University, Indianapolis, IN USA; Indiana Center for Vascular Biology and Medicine and VC-CAST Signature Center, Indianapolis, IN USA; Roudebush Veterans Affairs Medical Center, Indiana University, Indianapolis, IN USA; Division of Pulmonary and Critical Care Medicine, Indiana University, Indianapolis, IN USA

**Keywords:** Adipose stromal cells, ARDS, Endothelial barrier, Apoptosis

## Abstract

**Background:**

Acute Respiratory Distress Syndrome (ARDS) is a condition that contributes to morbidity and mortality of critically ill patients. We investigated whether factors secreted by adipose stromal cells (ASC) into conditioned media (ASC-CM) will effectively decrease lung injury in the model of lipopolysaccharide (LPS)-induced ARDS.

**Methods:**

To assess the effect of ASC-CM on ARDS indices, intravenous delivery of ASC and ASC-CM to C57Bl/6 mice was carried out 4 h after LPS oropharyngeal aspiration; Evans Blue Dye (EBD) was injected intravenously 1 h prior to animal sacrifice (48 h post-LPS). Lungs were either fixed for histopathology, or used to extract bronchoalveolar lavage fluid (BALF) or EBD. To assess the effect of ASC-CM on endothelial barrier function and apoptosis, human pulmonary artery endothelial cells were treated with ASC-CM for 48-72 h.

**Results:**

ASC-CM markedly reduced LPS-induced histopathologic changes of lung, protein extravasation into BALF, and suppressed the secretion of proinflammatory cytokines TNFα and IL6. White Blood Cells (WBC) from BALF of LPS-challenged mice receiving ASC-CM had decreased reactive oxygen species (ROS) generation compared to WBC from LPS-challenged mice receiving control media injection. Treatment of pulmonary endothelial monolayers with ASC-CM significantly suppressed H_2_O_2_-induced leakage of FITC dextran and changes in transendothelial resistance, as well as gap formation in endothelial monolayer. ASC-CM exposure reduced the percentage of endothelial cells expressing ICAM-1, and suppressed TNFα-induced expression of E-selectin and cleavage of caspase-3. ASC-CM reduced the endothelial level of pro-apoptotic protein Bim, but did not affect the level of Bcl-2, Bad, or Bad phosphorylation.

**Conclusions:**

Factors secreted by ASC efficiently reduce ARDS indices, endothelial barrier hyperpermeability, and activation of pro-inflammatory and pro-apoptotic pathways in endothelium.

## Background

ARDS is a serious condition resulting in respiratory failure and increased morbidity and mortality of critically ill patients [1,2]. ARDS can be triggered by a variety of pulmonary causes, such as viral infection, pneumonia, aspiration of gastric content, lung contusion, as well as non-pulmonary causes including sepsis, burn injury, multiple trauma, and acute pancreatitis [3]. The pathogenesis of ARDS includes inflammation of the lung parenchyma, infiltration of neutrophils into the airspaces, oxidative stress, disruption of the endothelial and epithelial barriers, damage to the epithelial lining and subsequent lung fibrosis [4]. Despite the fact that the mechanisms contributing to the pulmonary failure are well delineated, more than 20 years of clinical trials show that approaches aiming at the separate components of pathogenesis fail to affect mortality [5]. Currently, ARDS treatment remains primarily supportive. This situation highlights a need for a novel complex therapy, which would both limit the pathogenic mechanisms of ARDS and facilitate lung repair [6,7]. Cell therapy with adult mesenchymal stromal cells (MSC) may provide such multi-directional therapeutic action, as mesenchymal stromal cells exert pleiotropic effects, including anti-inflammatory and regenerative activities [8].

Initial studies exploring the effects of MSC on lung injury in rodents and explanted human lungs utilized bone marrow-derived mesenchymal stromal cells (MSC). These studies have shown that MSC suppressed lung injury in LPS- [9-11], *Escherichia coli*- [12], cecal ligation/puncture- [13,14], and bleomycin-induced [15] lung injury models. More recently, there has been increased interest in investigating MSC derived from adipose tissue (ASC) as a therapy for ARDS. The potential advantage of using ASC is their rapid isolation in therapeutic amounts via enzymatic digestion of the autologous adipose tissue harvested by liposuction, unlike MSC, which typically require weeks of propagation for a similar cell yield [16]. Similar to MSC, ASC are effective in inhibiting lung damage in ARDS in LPS- [17-20], cecal ligation/puncture- [21], ventilation- [22] and ischemia-reperfusion-induced [23] lung injury models.

While some studies have reported relatively high level of stem cell engraftment (ranging from 9-16% of cells administered intravenously) [24] and trans-differentiation into pulmonary cells [25], most studies emphasize stem cell-mediated anti-inflammatory effects along with stimulation of phagocytosis and bacterial clearance [9-13,15]. These effects have been attributed to secreted soluble factors from stem cells, and more recently to mitochondrial transfer from stem to host cells [26]. How effective are secreted soluble factors compared to the physical presence of ASC in ARDS therapy is an important question to answer, as the ability to use of-the-shelf conditioned media for therapeutic purposes will have obvious advantages compared to the use of banked or autologous cells.

In the current study, we compared the effects of ASC and ASC-CM on lung injury in a murine model of LPS-induced ARDS and analyzed the effects of ASC-CM on endothelium *in vitro*. The results of our study provide insight into the mechanisms of ASC beneficial action on inflamed lung, and have implications for the development of new cell-free therapy.

## Materials and methods

### Materials

*E. coli* LPS 0127:B8 with the lot activity of 3,000,000 U/mg, and 40 kDa fluorescein isothiocyanate (FITC)-dextran were purchased from Sigma (St. Louis, MO). The antibody recognizing VE-cadherin was from Cayman Chemical (Ann Harbor, MI); antibody to cleaved caspase-3 was from Cell Signaling (Beverly, MA); antibodies to Bcl-2 and Bad were from Santa Cruz Biotechnology (Dallas, TX); antibodies to phospho-Bad and Bim were from Cell Signaling (Beverly, MA), antibody to β-actin was from Sigma (St. Louis, MO). Carboxy-dichlorofluorescein diacetate (carboxy-DCFH-DA), and all reagents used for immunofluorescent staining were obtained from Invitrogen (Carlsbad, CA). All reagents for Flow Cytometry were from BD Biosciences (San Jose, CA).

### Animals

Male C57BL/6 mice were purchased from Harlan (Indianapolis, IN). All animal procedures were approved by Indiana University Institutional Animal Care and Use Committee and conformed to the requirements of Animal Welfare Act.

### Cell culture

All procedures for collecting human adipose tissue were approved by the Indiana University School of Medicine Institutional Review Board. Human ASC (hASC) were isolated from human subcutaneous adipose tissue samples obtained from liposuction procedures as previously described [27] and used at passage 3. Human pulmonary artery endothelial cells (HPAEC) were purchased from Lonza (Walkerville, MD) and used at passages 5–8. Murine ASC (mASC) were isolated from subcutaneous fat from the hip area using a similar procedure. Briefly, fat was excised from the anesthetized animal, minced and digested with 2 mg/ml collagenase type 1 (Worthington) at 37°C. Digests were centrifuged at 300 g to separate floating adipocytes. The pellet containing stromal vascular fraction was re-suspended in basal medium (EBM2 (Lonza)) supplemented with 5% FBS, filtered through 100 μ nylon filter, and centrifuged again at 300 g. Cells were re-suspended in complete growth medium (EGM2-MV (Lonza)), allowed to adhere to plastic, and propagated in EGM2-MV until 3rd passage at 37°C in a humidified atmosphere of 5%CO_2_-95% air. Before injection, cells were trypsinized and re-suspended in EBM2 at a concentration of 3 × 10^6^cell/mL.

### Flow cytometry

mASC passage 3 were harvested, counted with hemocytometer, and incubated for 20 min on ice with fluorophore-labeled anti-Sca-1 (positive selection marker), anti-CD31 and anti-CD45 (negative selection markers) IgG. Human ASC were tested for expression of CD13, CD73, CD90, CD105 (positive selection markers), CD31, and CD45 markers. Corresponding IgG were used as isotype controls. Flow cytometry was performed using a Calibur flow cytometer and Cell QuestPro software (BD).

### Generation of conditioned media

The composition of the media used for ASC-CM preparation was chosen depending on the nature of the following assay. Conditioned media for the assessment of the hASC-CM effects on endothelial permeability had to support HPAEC endothelial barrier over the pre-incubation period, and therefore was generated using complete growth media EGM2-MV. 50-60% confluent ASC were incubated with fresh EGM2MV 0.2 mL/cm^2^; media was collected 24 h later, and kept frozen until future analysis. Conditioned media for animal injections had to be FBS-free, and therefore was generated from mASC using basal EBM2 with 0.5% C57BL/6 mouse serum (Innovative Research, Novi, MI). When mASC-CM was generated, cells were allowed to reach same density as in the experiment when mASC were expanded for injection. Generated mouse ASC-CM (11 ml per 10^6^ cells) was concentrated 10 times with 3 kDa cut-off filter (Amicon, Billerica, MS) to render 1.1 ml of product per 10^6^ cells. mASC-CM was kept frozen until injection into animals. With this concentration, injection of 0.2 ml of mASC-CM is equivalent to the injection of factors generated by ~ 180 000 ASC. Corresponding base media (EBM2 with 0.5% C57BL/6 mouse serum) was subjected to identical manipulations and used for control injections in ASC-CM experiment.

### Assessment of ARDS indices

LPS (2 mg/kg of body weight) or an equal volume of saline was delivered to isoflurane-anesthetized 20-25 g C57BL/6 mice by oropharyngeal aspiration (OA), as described in [20]. Body temperature assessment was performed in alert animals using a rectal probe (YSI Life Sciences, Yellow Springs, OH).

In ASC experiments, either 360,000 murine ASC re-suspended in basal media EBM2 (passage 3) or equivalent volume of EBM2 (0.1 mL) were injected into tail vein 4 h after LPS administration. In ASC-CM experiments 0.2 mL of concentrated ASC-CM or corresponding control media were injected. Twenty-four or 48 h later, animals were sacrificed for analysis. For the assessment of vascular leak and lung inflammation, EBD- albumin conjugate (0.5% EBD/ 4% BSA solution in saline) was administered in the tail vein (30 mg/kg) 1 h prior to experiment termination. The chest cavity was opened under anesthesia and blood was sampled by cardiac puncture to determine level of circulating EBD. After nicking left atrium and severing abdominal aorta and vena cava, lungs were washed from blood by injecting saline via the right ventricle. Bronchoalveolar lavage fluid (BALF) was obtained by flushing lungs via trachea with 3 mL ice-cold PBS. Lungs were excised and homogenized in formamide to extract EBD (18 h, 60°C); optical density was determined at 620 and 750 nm. Extravasated EBD concentration was calculated using a standard curve and normalized to plasma EBD level. BALF was centrifuged at 600 g to sediment cells; supernatant was snap-frozen for future ELISA and protein content analyses. Pellet was subjected to red cell lysis; remaining cells were separated by cytospin and stained with Diff Quick staining kit (ThermoFisher Scientific, Waltham, MA). Cells were identified under Nikon microscope 40x objective; total of 300 cells were counted on each slide. For immunohistochemistry, lungs were perfused with 4% formaldehyde/agarose solution, embedded in paraffin, sectioned and analyzed with Nikon microscope (10x magnification objective).

### ELISA analyses

Mouse Quantikine ELISA kits were from R&D Systems (Minneapolis, MN). Undiluted BALF stored at −80° was used to determine concentrations of TNFα, IL-6, IL-10, MIP-2, and VEGF; all analyses were run in duplicate. Cytokine content in BALF was determined by multiplying cytokine concentration (pg/mL) by the exact volume of BALF obtained from a specific animal (approximately 3 mL).

### Assessment of ROS generation

The method for the measurement of oxidative activation of neutrophils was based on the ROS-dependent oxidation of carboxy-DCFH-DA to carboxy-DCF [28]. Cells from BALF of LPS- and LPS/ASC-CM treated mice were placed in wells of 96-well plate (60,000 cells per well), pre-loaded with 12.5 μg/ml carboxy-DCFH-DA, and then stimulated with 1 μg/ml LPS for 20 h. Wells with no cells containing the same concentration of dye were used as controls. Fluorescence was read in the FITC channel of fluorometer/plate reader. All assays were run in triplicate.

### Measurement of transendothelial permeability

Permeability of HPAEC monolayers for FITC-dextran was measured using 0.4 μ polyester trans-well inserts (Costar) as described in [29]. Briefly, HPAEC were plated on collagen-coated inserts, whereas ASC or NHDF were plated on the bottom of wells. HPAEC were allowed to reach confluency. Alternatively, HPAEC were pretreated with un-concentrated ASC or NHDF conditioned media mixed with EGM2MV (1:1) for 72 h. Media were changed to EBM-2 (Lonza) 1 h prior to FITC-dextran loading. FITC-dextran was added to the top chamber to a final concentration 1.75 mg/mL; immediately after, monolayers were stimulated with 0.25 mM H_2_O_2_. After 2 h, media was sampled from the bottom chamber and analyzed for FITC-dextran fluorescence. All analyses were run in quadruplicate.

Transendothelial electrical resistance (TER) was measured using the highly sensitive biophysical assay with an electrical cell-substrate impedance sensor (ECIS) (Applied Biophysics, Troy, NY) as described previously [29]. HPAEC were plated on gold microelectrodes in EGM2MV; then exposed to the un-concentrated conditioned media/EGM2MV mixture (1:1) for 72 h. At the end of pre-incubation period, resistance of monolayers reached 1000–1200 Ohms, evident of monolayer confluence. Media were changed to basal media 1 h prior to the beginning of the TER recording. 30 min after the start of recording, monolayers were stimulated with 0.25 mM H_2_O_2_.

### HPAEC imaging

For immunofluorescence experiments, HPAEC monolayers were plated on gelatin-coated coverslips and grown to confluence. Cells were exposed to the un-concentrated conditioned media mixed with EGM2-MV (1:1) for 72 h; media were changed to EBM2 1 h prior to the beginning of the experiment. After H_2_O_2_ stimulation, cells were fixed, permeabilized and stained with VE-cadherin-specific antibodies and Alexa594-conjugated phalloidin. Coverslips were viewed and photographed using Nikon fluorescent microscope (40x objective).

### Assessment of VCAM, ICAM-1, and E-selectin expression

HPAEC grown in 6 well plates were exposed to the un-concentrated conditioned media mixed with EGM2-MV (1:1) for 72 h; media were changed to EBM2 1 h prior to the beginning of the experiment. 4 h after TNFα/vehicle control stimulation, cells were harvested and incubated with fluorescently labeled IgG against CD54/ICAM-1 (eBioscience, San Diego, CA), CD62E/E-selectin (Biolegend, San Diego, CA), and CD106/VCAM (BD) on ice for 30 minutes. Labeled cells were analyzed with Guava 8HT flow cytometer ((EMD Millipore, Billerica, MA).

### Western immunoblotting

HPAEC grown in 12-well plates were exposed to the un-concentrated conditioned media mixed with EGM2-MV (1:1) for 48-72 h; media were changed to EBM2 1 h prior to the beginning of the experiment. After 4 h stimulation with TNFα, cells were rinsed with ice-cold PBS and lysed with 1% SDS containing buffer. Protein extracts were separated on 4-20% gels and transferred to nitrocellulose membrane. After staining with specific antibodies, membranes were developed and scanned using BioRad imaging system.

### Statistical analysis

Quantitative data are presented as mean ± SEM, unless otherwise indicated. Statistical analysis was performed by t-test, t-test with Welch’s correction (unequal variance), One-way ANOVA with Tukey post-hoc or Repeated Measures One-way ANOVA using GraphPad Prizm6 or Origin 8.0 software. Shapiro-Wilk test was used to confirm that data were drawn from a normally distributed population. A probability value of < 0.05 was considered statistically significant.

## Results

### Flow cytometric analyses of ASC surface markers

**Figure 1:**
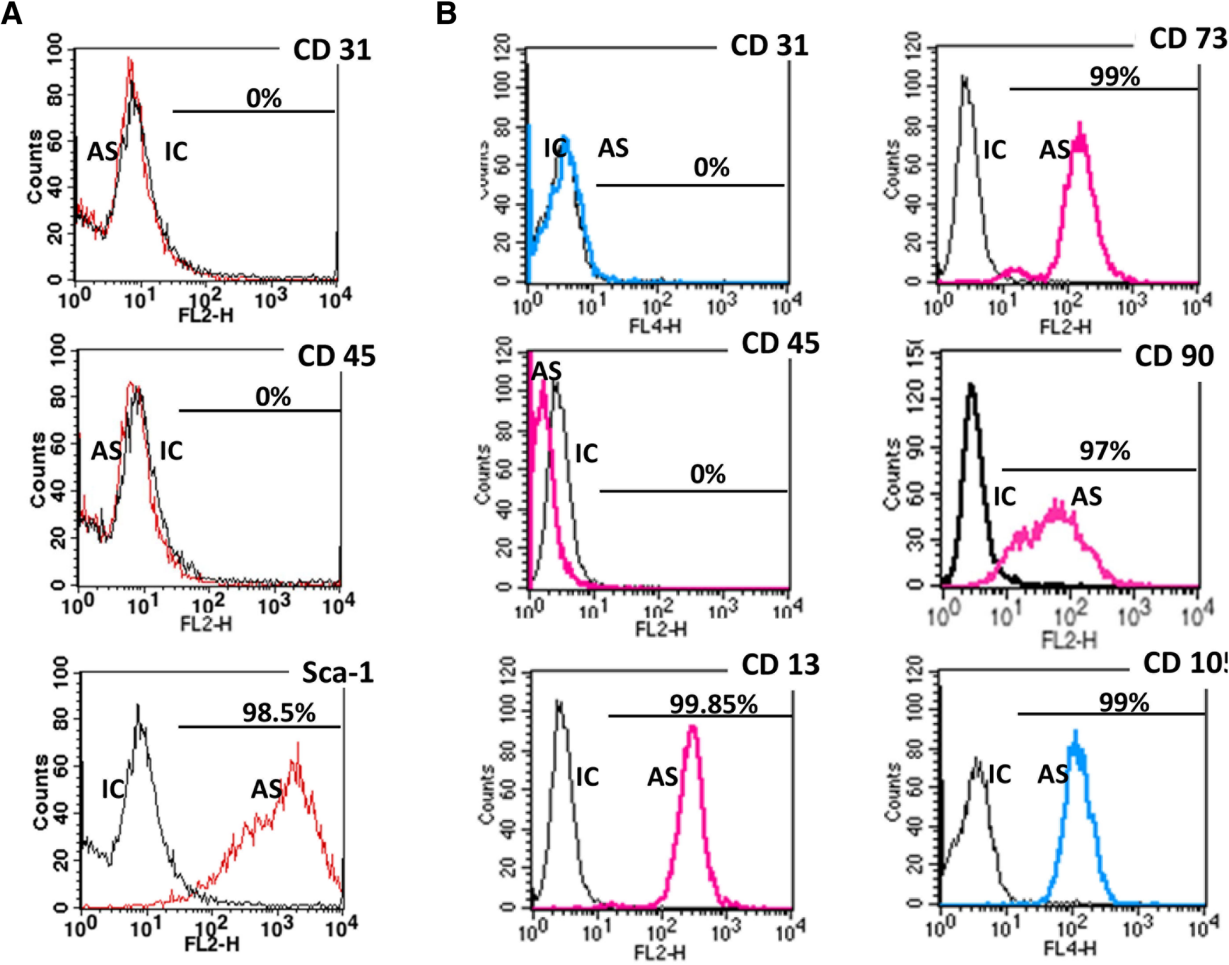
**Surface marker expression of mASC (A) and hASC (B).** IC-isotype controls, AS- antigen-specific antibodies. As expected, the majority of mASCs expressed the surface antigen of mesenchymal stem cells Sca-1, and was negative for the hematopoietic stem cell marker (CD45) and endothelial marker (CD31). The majority of hASC expressed stromal markers CD13, CD73, CD90, and CD105, and was negative for CD31, CD45.

Both murine and human ASC used in our study were produced by propagation of the cell fraction adherent to plastic under standard culture conditions. mASC and hASC demonstrated typical size, spindle shape, and morphology in culture consistent with adipose stem cells. mASC as well as hASC were evaluated with respect to their surface marker phenotype at passage 3. mASC were positive for Sca-1, one of the markers routinely used to characterize mesenchymal stem cells of murine origin [30], and negative for the endothelial cell marker CD31 and the marker of cells of hematopoietic origin CD45 (Figure 1A). Similarly, analysis of hASC at passage 3 demonstrated positive staining for the stromal markers CD13, CD73, CD90 and CD105 and negative staining for CD31 and CD45 (Figure 1B).

### Study design

**Figure 2:**
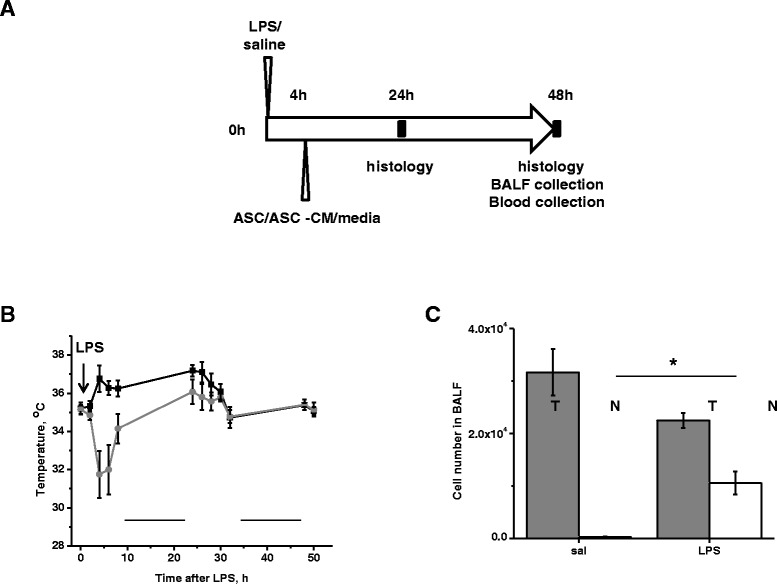
**Administration of ASC/ASC-CM is scheduled at the onset of ARDS. A**. Timeline of the *in vivo* study design. **B**. Body temperature measured in mice receiving saline (black) or LPS (grey) via oropharyngeal aspiration (N = 3-4 per group). First time point of the day was recorded at 8 a.m. Black lines indicate periods of dark. **C**. Total WBC (T, dark grey) and neutrophil (N, white) count in BALF of mice receiving saline or LPS (N = 3 per group). BALF was collected 4 h post-saline/LPS administration. T-test with Welch’s correction was used to assess the differences between analyzed groups.

LPS or saline were delivered into lungs through oropharyngeal aspiration (Figure 2A). Intravenous ASC, ASC-CM or control media injections were performed 4 h after LPS delivery. This protocol was designed to schedule the ASC administration concurrently with the onset of ARDS, identified by peak of hypothermia at 4 h following LPS administration (Figure 2B) and by the first presence of neutrophil infiltration in BALF (Figure 2C).

### Effect of ASC or ASC-CM on lung histopathology following LPS administration

**Figure 3:**
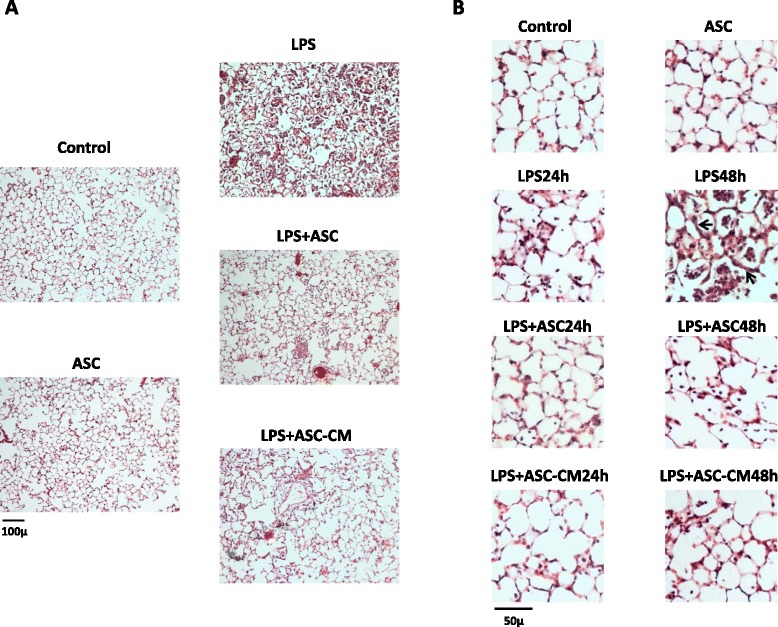
**mASC and mASC-CM markedly reduce LPS-induced lung injury. A**. Histopathology of lung 48 h post-saline/LPS shown at 10X magnification. **B**. High power fields at the time points indicated. Note the thickening of alveolar wall and the appearance of cellular infiltrate in the airspaces in response to LPS (arrows).

Hematoxylin and eosin staining of the lung sections revealed no apparent signs of inflammatory response in saline-treated lungs of mice which received ASC or control media injection (Figure 3A,B). LPS triggered lung inflammation, which was evident at 24 h and severe at 48 h. In mice receiving control media injection, LPS caused marked infiltration of neutrophils and red blood cells into the lung interstitium and airspaces, as well as swelling of the alveolar walls. In contrast, systemic delivery of ASC or ASC-CM reduced septum thickening and suppressed accumulation of cells and debris in the alveolar sacs (Figure 3).

### ASC and ASC-CM effects on lung permeability and neutrophil infiltration

**Figure 4:**
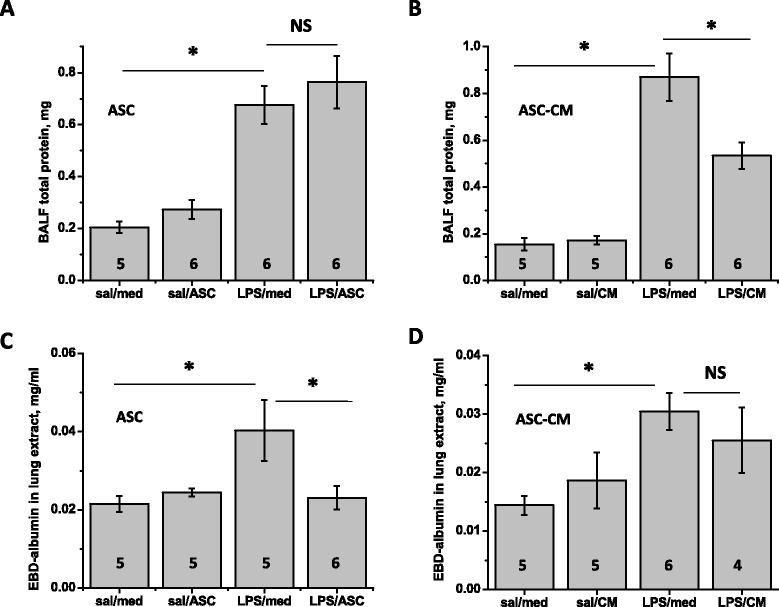
**mASC and mASC-CM effect on epithelial and endothelial permeability.** Protein in BALF collected 48 h post-injection was significantly reduced by mASC-CM **(B)**, but not mASC **(A)**. Conversely, EBD content in lung was significantly reduced by mASC **(C)**, but not mASC-CM **(D)**. One-way Anova with Tukey post-hoc was used to assess the significance of differences between the groups; number of animals is indicated.

Assessment of total protein content in BALF showed a significant increase of protein level in response to LPS administration (Figure 4 A,B). While ASC treatment had no effect on LPS-induced protein increase in BALF, ASC-CM treatment markedly suppressed the LPS-induced protein increase 48 h post-injection. On the contrary, ASC were able to suppress LPS-induced increase in EBD extravasation (Figure 4C), whereas ASC-CM effect was not significant (Figure 4D).

**Figure 5:**
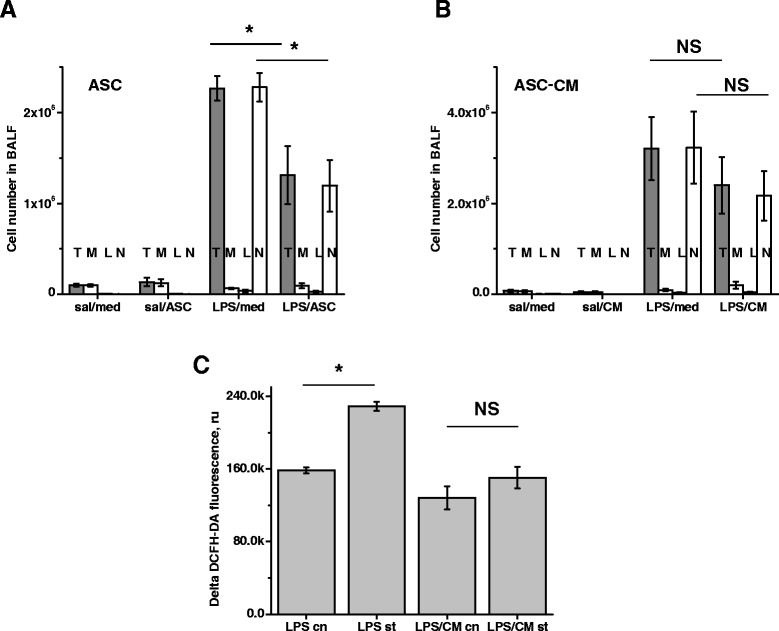
**mASC and mASC-CM effect on neutrophil numbers and activity.** Total WBC (T, grey) and PMN (N, white) counts in BALF were significantly reduced by mASC **(A)**, but not mASC-CM **(B)**. The ratio between macrophages (M), lymphocytes (L) and neutrophils (N) in BALF of LPS-treated mice was not significantly affected by either experimental treatment. N = 5-6 animals per group. **(C)** WBC from BALF of LPS-challenged mice (N = 6) were subjected to vehicle (LPC cn) or 1 μg/ml LPS stimulation (LPS st). In parallel, WBC from BALF of LPS/ASC-CM-treated mice (N = 6) were subjected to vehicle control (LPS/CM cn) or LPS stimulation (LPS/CM st). LPS-induced ROS generation by WBC from BALF of LPS/ASC-CM mice was significantly lower comparing to WBC from BALF of LPS/media mice. T-test was used to analyze the significance of differences between groups.

Assessment of total WBC count in BALF showed that the number of cells in airspaces increased dramatically in response to LPS stimulation (Figure 5A,B). Whereas resident lung macrophages comprised the majority of cells in BALF from saline lungs, neutrophils were the dominant cell type in BALF from LPS-challenged lungs. Although ASC or ASC-CM treatment did not shift the overall neutrophil/macrophage balance (Figure 5A,B), a significant suppression of total WBC count was evident in LPS mice receiving ASC injection (Figure 5A). A similar trend was observed in mice receiving ASC-CM, but did not reach significance at 48 h post-injection (Figure 5B). To further characterize possible mechanisms of ASC-CM-mediated lung injury limitation, we compared the ability of BALF WBC, obtained from LPS- or LPS/ASC-CM-receiving mice, to generate ROS. The method for the measurement of neutrophils oxidative activity was based on ROS-dependent oxidation of DCFH-DA (non-fluorescent) to highly fluorescent DCF. Isolated BALF WBC were tested for their abilities to oxidize DCFH-DA in response to LPS stimulation in vitro. We observed that WBC from mice receiving LPS can be further induced by LPS stimulation, whereas WBC from mice receiving LPS/ASC-CM no longer respond to LPS stimulation (Figure 5C), suggesting that mice exposure to ASC-CM leads to lung recruitment of neutrophils with a reduced potential for oxidative response.

### ASC and ASC-CM effects on accumulation of pro- and anti-inflammatory cytokines and pro-angiogenic factor in BALF

**Figure 6:**
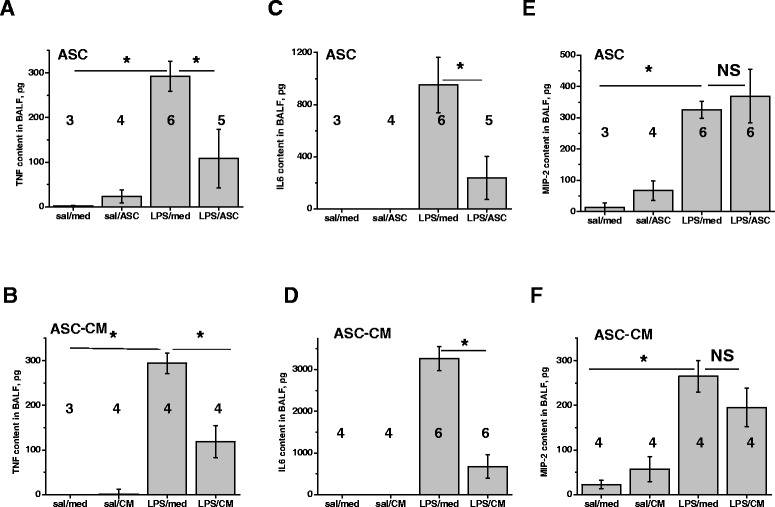
**mASC and mASC-CM effect on pro-inflammatory cytokines in BALF.** mASC and mASC-CM reduced the level of TNFα and IL6 **(A-D)**, but not MIP-2 **(E-F)**. One-way Anova with Tukey post-hoc was used to assess the significance of differences between the groups; number of animals is indicated.

**Figure 7:**
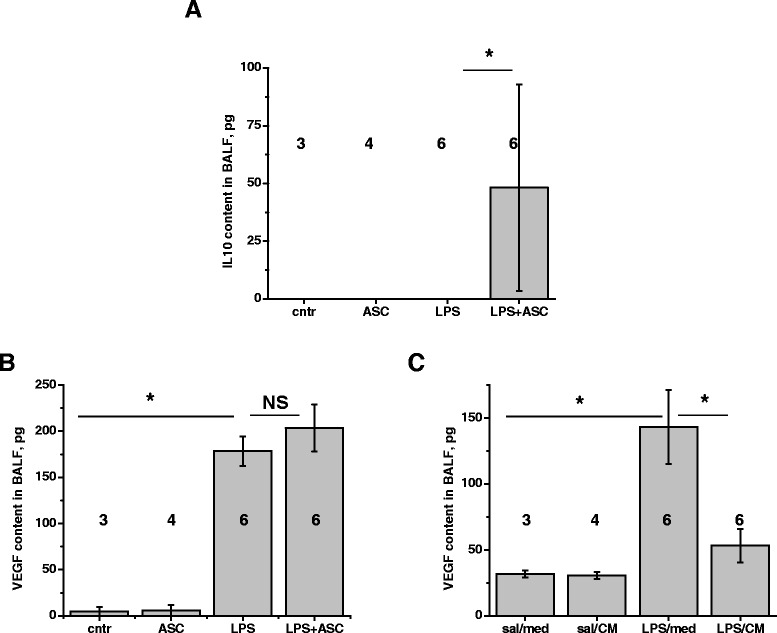
**mASC and mASC-CM effect on the level of anti-inflammatory cytokine IL10 and pro-angiogenic factor VEGF.** IL10 was detected in BALF of LPS-challenged mice injected with mASC **(A)**, but not mASC-CM (data not shown). LPS-induced increase in VEGF level was reduced in response to mASC-CM injection **(C)**, but not mASC injection **(B)**. One-way Anova with Tukey post-hoc was used to assess the significance of differences between the groups; number of animals is indicated.

To determine the effect of ASC and ASC-CM on inflammation progression/resolution, we assessed the level of the pro-inflammatory cytokines TNFα, IL6, and MIP-2, and anti-inflammatory IL10 in BALF. LPS caused marked increase in the level of TNFα, IL6 and MIP-2 (Figure 6A-F). Forty-eight hours after cell or conditioned media injection, LPS-induced increase in TNFα and IL6 levels was suppressed by both agents, whereas the increase in MIP-2 level was not affected significantly. The level of anti-inflammatory IL10 remained below detection in control mice as well as mice challenged with LPS. Significant increase in BALF IL10 content was observed in response to ASC (Figure 7A), but not ASC-CM (data not shown). Interestingly, the LPS-induced level of VEGF in BALF was non-significantly increased in ASC-treated mice, but markedly suppressed in ASC-CM-treated mice (Figure 7B-C).

### ASC and ASC-CM effects on endothelial permeability in vitro

**Figure 8:**
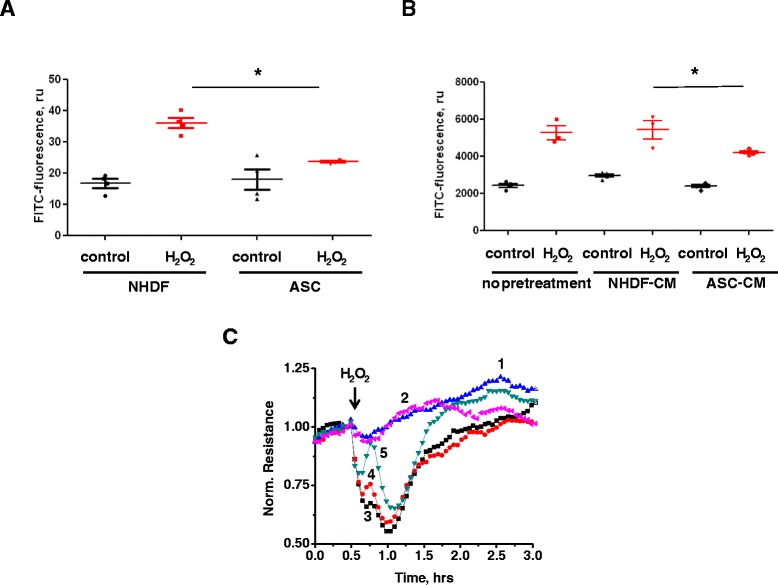
**hASC and hASC-CM preserve HPAEC transendothelial permeability.** HPAEC were grown on polyester inserts **(A-B)** or on gold electrodes of ECIS arrays **(C)** in the absence/presence of NHDF/hASC **(A)**, or NHDF/hASC conditioned media **(B-C)**. HPAEC were removed from the contact with cells/media; then stimulated with 250 μM H_2_O_2._ T-test with Welch’s correction was used to assess the differences between analyzed groups. NHDF and NHDF-CM were used for comparison as a non-stem cell/conditioned media control. **(C)** Shown are the means of 3 parallel recordings for each pretreatment/stimulation: 1) unstimulated HPAEC pretreated with NHDF-CM; 2) unstimulated HPAEC pretreated with hASC-CM; 3) stimulated with H_2_O_2_ HPAEC which were not pretreated with CM; 4) stimulated with H_2_O_2_ HPAEC which were pretreated with NHDF-CM; 5) stimulated with H_2_O_2_ HPAEC pretreated with hASC-CM. Repeated measures One-way ANOVA showed significant differences between response of NHDF-CM-pretreated cells and response of ASC-CM pretreated cells. Preincubation of endothelial monolayers with hASC or hASC-CM reduced H_2_O_2_-induced hyperpermeability of HPAEC to FITC-dextran **(A-B)** or to ions **(C)**.

**Figure 9:**
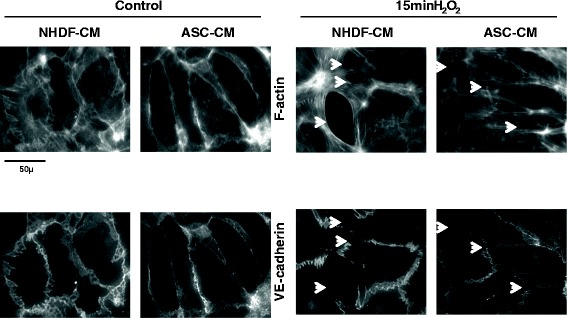
**hASC-CM preserves microscopic HPAEC monolayer integrity.** Preincubation of endothelial monolayers with hASC-CM suppressed H_2_O_2_-induced rearrangement of F-actin cytoskeleton and disintegration of peripheral adherens junctions. Arrows indicate the gaps appeared in previously intact monolayer in response to H_2_O_2_.

To further analyze mechanisms underlying ASC and ASC-CM effects on lung permeability, we directly assessed ASC/ASC-CM effects on monolayer barrier function of well-characterized human pulmonary endothelial cell line HPAEC. Since recently it was suggested that syngeneic ASC therapy has more potent effect than xenogeneic therapy [20], we tested the effect of hASC/hASC-CM on the permeability of human pulmonary endothelium. First, HPAEC monolayers were grown on collagen-coated polyester inserts in the presence of hASC or NHDF (as a non-stem control) in the lower chamber. Prior to analysis, inserts were transferred to fresh wells to avoid the possibility of direct ROS scavenging by hASC/NHDF. The top chamber was loaded with FITC-dextran, and monolayers were stimulated with H_2_O_2_, the edemagenic product of the neutrophil oxidative burst. We observed marked HPAEC barrier dysfunction in response to H_2_O_2_, which was significantly attenuated in monolayers grown in the presence of hASC (Figure 8A). We next pretreated HPAEC monolayers with either hASC-CM or NHDF-CM. Similar to HPAEC grown in the presence of hASC, HPAEC grown in the presence of hASC-CM responded to H_2_O_2_ with less barrier dysfunction (Figure 8B); this stabilizing effect was not seen with NHDF-CM. To further characterize the hASC-CM-mediated stabilization of HPAEC barrier, we assessed transendothelial electrical resistance (TER). HPAEC grown on gold electrodes were pretreated with hASC-CM or NHDF-CM; then stimulated with H_2_O_2_. Control monolayers responded to H_2_O_2_ with marked reduction of TER, which was restored within 3 h. NHDF-CM-pretreated monolayers displayed a similar response curve, whereas hASC-CM-pretreated monolayers had a significantly attenuated barrier dysfunction in response to H_2_O_2_ (Figure 8C). Immunofluorescent microscopy of NHDF-CM-pretreated HPAEC showed that H_2_O_2_ caused marked rearrangement of the actin cytoskeleton along with distortion of junctional organization and gap formation in endothelial monolayers (Figure 9, arrowheads). hASC-CM-pretreated HPAEC displayed less severe changes in response to H_2_O_2,_ with attenuated gap formation.

### ASC-CM effect on the expression of ICAM-1 and E-selectin in endothelium

**Figure 10:**
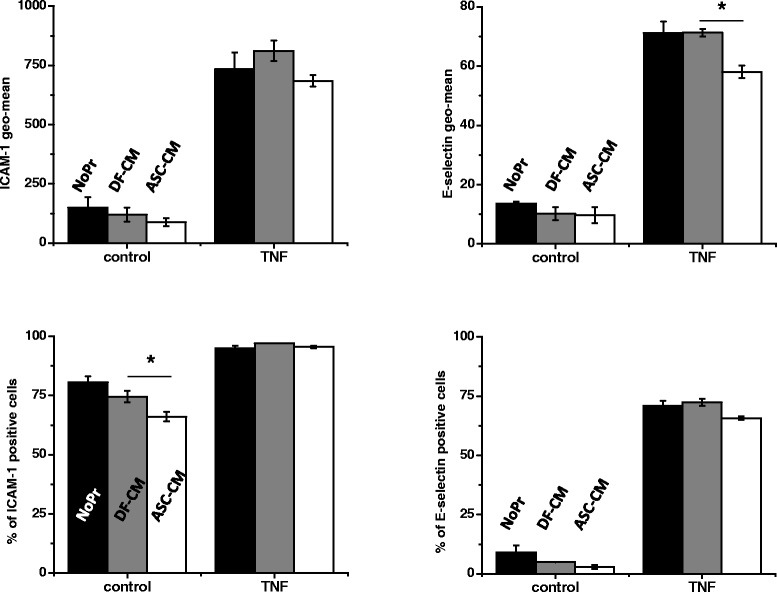
**ASC-CM suppresses ICAM-1 and E-selectin expression by HPAEC.** HPAEC were treated with vehicle control (NoPr, black bars), NHDF-CM (DF-CM, grey bars), and hASC-CM (ASC-CM, white bars), and then stimulated with vehicle or 1 ng/ml TNFα (4 h). Harvested cells were analyzed for the surface expression of ICAM-1 and E-selectin. Experiments with independent treatment/stimulation were repeated in triplicate. T-test was used to assess the differences between analyzed groups. Exposure to hASC-CM lowers the percentage of ICAM-1-expressing quiescent HPAEC and reduces the level of E-selectin expression in TNFα-stimulated HPAEC.

To ascertain whether hASC-CM anti-inflammatory effect is dependent on the suppression of endothelial expression of leucocyte receptors, we assessed the level of expression of VCAM, ICAM-1, and E-selectin in naïve and TNFα-stimulated HPAEC. Low percentage of unstimulated cells appeared positive for VCAM (data not shown) and E-selectin, whereas expression of ICAM-1 was detected in at least 70% cells (Figure 10). TNFα caused dramatic increase in the percentage of VCAM- (data not shown) and E-selectin-positive cells, also increasing ICAM-1 expression evident by the shift in ICAM-1 geo-mean value. HPAEC exposed to hASC-CM did not show significant difference in the expression of VCAM either in absence or presence of TNFα (data not shown). Percentage of ICAM-positive cells detected in the absence of TNFα was significantly suppressed by hASC-CM treatment; however, this effect was not detected in the presence of TNFα. On the contrary, E-selectin expression (evident by geo-mean) was significantly suppressed in the presence of TNFα.

### hASC-CM effect on the activation of pro-apoptotic pathways in endothelium

**Figure 11:**
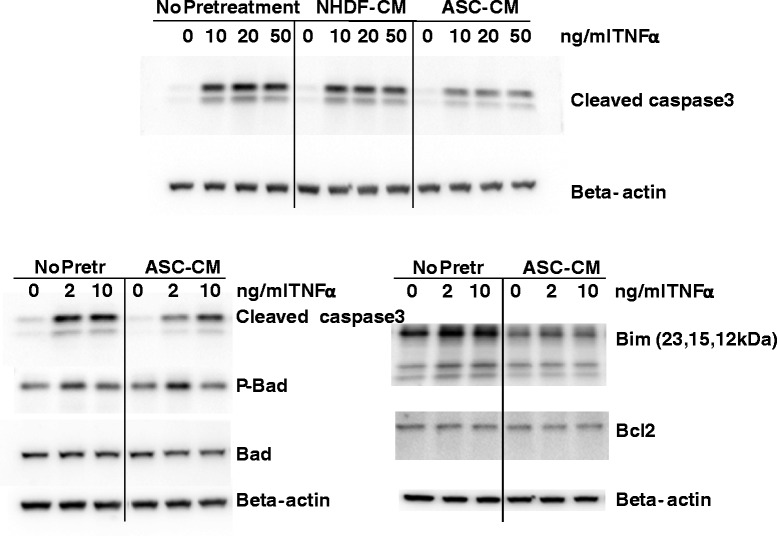
**ASC-CM suppresses activation of pro-apoptotic pathways in HPAEC.** HPAEC were treated with vehicle control, NHDF-CM, and hASC-CM, and then stimulated with indicated concentration of TNFα (4 h). Cell lysates were analyzed with antibodies to cleaved caspase 3, Bad, phospho-Bad, Bim, and Bcl2. β-actin staining was used as loading control.

To elucidate whether anti-inflammatory effects of hASC-CM are mediated by the suppression of pro-apoptotic changes in endothelium, we assessed the level of caspase-3 cleavage in response to TNFα (Figure 11). TNFα induced cleavage and activation of caspase-3 evident at 4 h. HPAEC pretreated with hASC-CM showed a marked reduction of cleaved caspase-3 level in response to TNFα. As caspases activation is known to be regulated by pro-apoptotic and anti-apoptotic members of Bcl2 family [31], we next investigated whether levels of these proteins are affected by hASC secreted factors. Levels of anti-apoptotic protein Bcl2 and pro-apoptotic protein Bad did not change in HPAEC preconditioned with hASC-CM. We did not observe consistent increase in Bad phosphorylation (evident of Bad deactivation) in response to hASC-CM pretreatment. However, exposure to hASC-secreted factors markedly reduced the level of pro-apoptotic protein Bim.

## Discussion

Here, we demonstrate for the first time that administration of ASC-CM reduces indices of lung injury in the LPS-induced ARDS model. Although autologous ASC can be easily isolated from liposuction material within hours of the patient admission to ICU [16], the ability to avoid this procedure and use an “off-the-shelf” therapeutic product could be a significant advantage for the treatment of critically ill patient. Another potential advantage of the use of conditioned medium is the absence of concerns associated with the possibility of cells homing toward pre-existing tumors [32] or causing thromboemboli [33].

Previously, we have demonstrated that ASC-CM administration is able to prevent and reverse brain hypoxic-ischemic injury [34]. Here, we assessed whether ASC-CM would exhibit an ability to prevent development of LPS-induced lung injury. Our study showed that a single administration of ASC-CM limited lung inflammatory histological changes, protein extravasation to airspaces, accumulation of inflammatory mediators TNFα and IL6 in BALF, and the ability of BALF WBC to generate ROS. One limitation of this study was that ARDS indices were analyzed via procedures requiring animal termination, which limits the assessment of precise kinetics of the response. We observed that ASC-CM-mediated suppression of LPS-induced neutrophil infiltration showed a trend parallel to ASC, yet did not reach significance at 48 h post-injection. We also did not detect the suppression of LPS-induced vascular leakage at this time point. We can only speculate whether the suppression of LPS-induced neutrophil infiltration and vascular leakage by ASC-CM is significant at the earlier time points in our model; however, it is to be expected that sustained retention of cells in the inflamed lung would elicit more profound effects than the bolus injection of the beneficial cell-secreted factors. Nonetheless, our data suggest that the ability of neutrophils to cause damage to the parenchyma and epithelium via oxidative burst is attenuated in the mice receiving ASC-CM. Moreover, our *in vitro* data show that the abilities of endothelium to resist H_2_O_2_-induced barrier dysfunction and TNF-induced pro-inflammatory and pro-apoptotic changes are enhanced by ASC secreted factors. We show here that the exposure of endothelium to ASC-CM lowers the level of pro-apoptotic protein Bim. All three Bim isoforms are known to induce apoptosis [35]. Bim plays an important role in Bax/Bak-mediated cytochrome C release and activation of caspase cascade [36]. We believe that down-regulation of pro-apoptotic proteins by ASC-secreted factors is a novel mechanism, contributing to alleviation of endothelial pathology and pulmonary vascular dysfunction. Whether down-regulation of pro-apoptotic proteins is observed in ARDS lungs in response to ASC or ASC-CM therapy, is a subject for future research.

In our in vivo model utilizing a single delivery of ASC-CM, secreted factors showed somewhat inferior potency compared to ASC, except for protein extravasation into BALF. Increased protein extravasation results from a leaky epithelial barrier, which is likely to be counteracted by ASC-secreted factors, similar to the effect shown on endothelium. Surprisingly, we did not detect a beneficial effect of ASC on LPS-induced protein extravasation. We assume that the host response to ASC engrafted into inflamed lung is likely to be different from the host response to a single delivery of ASC-secreted factors. Specifically, our results show that ASC-CM treatment causes marked reduction of LPS-induced VEGF level in BALF, whereas ASC treatment had little effect on this endpoint. VEGF secreted to airspace may facilitate long-term tissue repair, but also temporarily contribute to the compromise of barrier function. Therefore, the effect of ASC on ARDS may involve an interplay between the resident lung tissue responses and the infused ASC, with the need to analyze temporal factors for the optimization of therapy.

Recently, concentrated microvesicular fraction of the conditioned media from bone marrow-derived MSC was shown to alleviate LPS-induced lung injury [37]. However, to achieve an effect comparable to the effect of cell therapy, the vesicular fraction was concentrated from the media conditioned with approximately 4 times the amount of MSC used for injection. On the contrary, in our experiments the media was concentrated from only half the amount of ASC used for injection. These data suggests that either the microvesicle preparation process led to a partial loss of activity, or that bone marrow-derived MSC therapy relies to a larger extent on cell-to-cell transfer of bioactive material than on bioactive material secretion.

The effects of ASC therapy on lung pathology may include both cellular (e.g., mitochondrial transfer), as have been proposed [26] as well as paracellular (secreted factors) mechanisms. This study indicates that a single dose of factors secreted by ASC-CM is sufficient to limit several indices of ARDS. Future studies should investigate if continuous delivery of ASC-CM has increased efficacy in this and other models of ARDS.

## Conclusions

Systemic administration of ASC conditioned media diminishes LPS-induced lung injury by inhibiting protein extravasation into BALF, neutrophil inflammatory response, secretion of pro-inflammatory cytokines TNFα and IL6, and histopathological changes of the lungs. Exposure of endothelium to factors secreted by ASC attenuates endothelial barrier dysfunction and pro-inflammatory and pro-apoptotic changes. Suppression of pro-apoptotic pathways is likely to be mediated by the down-regulation of certain pro-apoptotic proteins in endothelium. Our findings provide novel insights into the paracrine mechanisms of ASC’s beneficial effects in ARDS.

## Abbreviations

ARDS: Adult respiratory distress syndrome
ASC: Adipose stromal cells
ASC-CM: ASC conditioned media
BALF: Bronchoalveolar lavage fluid
carboxy-DCFH-DA: Carboxy-dichlorofluorescein diacetate
EBM2: Endothelial basal media 2
EGM2-MV: Endothelial growth media 2 for microvascular cells
FITC-dexran: Fluorescein isothiocyanate-dextran
HPAEC: Human pulmonary artery endothelial cells
ICU: Intensive care unit
LPS: Lipopolysaccharide
MSC: Mesenchymal stromal cells
NHDF: Normal human dermal fibroblasts
ROS: Reactive oxygen species
TER: Transendothelial resistance
WBC: White blood cells
